# Exosomes in Neuroblastoma Biology, Diagnosis, and Treatment

**DOI:** 10.3390/life12111714

**Published:** 2022-10-27

**Authors:** Leila Jahangiri, Tala Ishola

**Affiliations:** 1 Division of Cellular and Molecular Pathology, Department of Pathology, Addenbrookes Hospital, University of Cambridge, Cambridge CB2 0QQ, UK; 2 School of Science and Technology, Nottingham Trent University, Clifton Site, Nottingham NG11 8NS, UK; 3 Department of Life Sciences, Birmingham City University, Birmingham B15 3TN, UK

**Keywords:** neuroblastoma, extracellular vesicles, exosomes, diagnosis, treatment

## Abstract

Neuroblastoma is an extracranial solid tumour of the developing sympathetic nervous system accounting for circa 15% of deaths due to cancer in paediatric patients. The clinical course of this cancer may be variable, ranging from aggressive progression to regression, while the amplification of MYCN in this cancer is linked to poor patient prognosis. Extracellular vesicles are a double membrane encapsulating various cellular components including proteins and nucleic acids and comprise exosomes, apoptotic bodies, and microvesicles. The former can act as mediators between cancer, stromal and immune cells and thereby influence the tumour microenvironment by the delivery of their molecular cargo. In this study, the contribution of extracellular vesicles including exosomes to the biology, prognosis, diagnosis and treatment of neuroblastoma was catalogued, summarised and discussed. The understanding of these processes may facilitate the in-depth dissection of the complexity of neuroblastoma biology, mechanisms of regression or progression, and potential diagnostic and treatment options for this paediatric cancer which will ultimately improve the quality of life of neuroblastoma patients.

## 1. Introduction

Neuroblastoma (NB), is an aggressive paediatric malignancy leading to death in children of mainly preschool age and this cancer accounts for a large fraction (i.e., 15%) of cancer-related mortality in children [1]. The specific age groups at diagnosis will vary from 0–18 months (infants), 18 months–<12 years (children), and adolescents (≥12) [1]. This cancer develops due to the dysregulation of the sympathetic nervous system and its cell of origin is thought to be within the neural crest. The clinical course of this cancer can vary from regression to refraction and relapse despite substantial treatment [2]. The main sites of presentation of NB include the abdomen, pelvis, neck and chest [3], while upon diagnosis, circa 50% of patients may display metastasis to the bone, lymph nodes, liver and bone marrow, bearing dismal prognostic predictions [4]. This heterogeneity will reflect risk groups, prognosis and treatment options and accordingly the international neuroblastoma risk group (INRG) has established a consensus on pre-treatment risk stratification based on differentiation status, histological properties, MYCN status, ploidy, age, segmental chromosomal alterations and stage among other factors [5,6]. Based on these characteristics, NB patients can be stratified into risk groups, including low, intermediate and high. Consistently, circa 20–30% of all NB cases bear MYCN amplification, equating to 40–50% of all high-risk cases, while the amplification of MYCN is the greatest predictor of poor prognosis, since the status of this oncogene correlated with disease aggression and resistance to treatment [5,6]. In addition to MYCN amplification, high-risk NB may be categorised into two other distinct molecular subgroups that display either an alternating length of telomeres (ALT) phenotype (circa 24% of high-risk cases) or upstream rearrangements with respect to the telomere reverse transcriptase (*TERT*) gene (circa 23–31% of high-risk cases) [7,8,9]. Consistently, the latter subgroup includes the rearrangements of chromosome 5p15.33 leading to an enhanced expression of *TERT* due to the juxtaposition of a strong enhancer into the coding sequence of this gene [7,8,9]. Interestingly, the common feature of all three subgroups mentioned is altered telomere maintenance mechanisms (TMMs) since the MYCN amplified subgroup may display TERT upregulation, while each subgroup is also associated with poor patient outcomes [10]. Mechanistically, telomerase uses a human RNA telomerase component, hTR to synthesise telomere DNA, while ALT, largely based on non-canonical homologous recombination, is linked to somatic alterations in Alpha thalassemia mental retardation-X linked (*ATRX*) gene in NB [11,12].

Finally, NB patients can also be categorised based on the pre-operative international neuroblastoma risk group staging system (INRGSS) which is based on the presence of image-defined risk factors (IDRF) and include L1, L2, M and MS stages, while the international neuroblastoma staging system (INSS) is largely based on the potential for resection and is categorised into stages 1–4 and 4S (reviewed in [5,6]). Accordingly, L1 is mainly amenable to resection, L2 is more prone to initial biopsy, neoadjuvant therapy and surgery, and MS is specific to children under the age of 18 months with a localised disease representation along with bone marrow (except for the cortical bone), liver and skin metastasis, while M comprises distant metastasis. INSS stages, on the other hand, include stages 1 and 2 which are locally resectable, and partially resectable with potential lymph node involvement, respectively [5,6]. Stage 3 disease crosses the midline, is generally deemed unresectable, and has spread to the lymph nodes contralaterally, while stage 4 involves distant metastasis. 4S has an upper limit of 12 months of age, where the tumour is confined to the area in which it arose, but some cells have spread to the skin, liver, or bone marrow [5,6].

**Figure 1:**
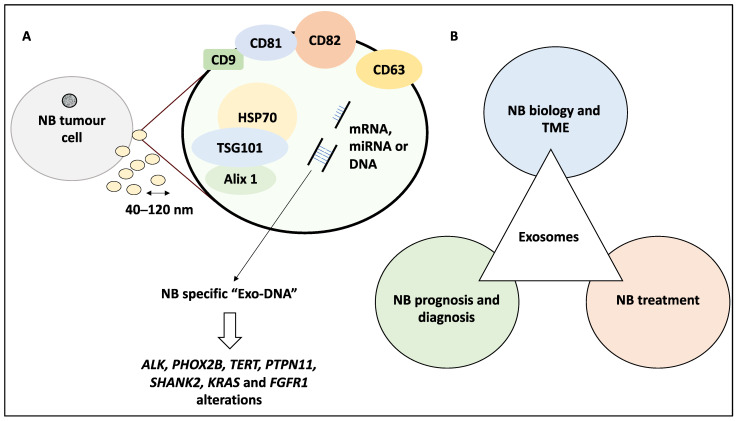
The general characteristics of exosomes derived from NB tumour cells and the objectives of this study. (**A**) Exosomes express markers including TSG101, HSP70, Alix 1, CD9, CD63, CD81, and CD82 and encapsulate molecular cargo including proteins, DNA, miRNA and mRNA among other molecules. Exo-DNA may represent an NB-tumour signature, cover a wide range of chromosomal DNA and can be used to screen for genetic alterations including *ALK*, *PHOX2B*, *TERT*, *PTPN11*, *SHANK2*, *KRAS* and *FGFR1*. (**B**) The objective of this study is to discuss the contribution of exosomes to NB biology, TME, prognosis, diagnosis and treatment.

Cells can communicate via cell-to-cell contact, secretion of molecules and extracellular vesicles (EVs). The latter has been classified into apoptotic bodies, microvesicles, and exosomes [13,14]. This classification is based on biogenesis routes and size variations from 400–4000 nm, 100–1000 nm and 40–120 nm for each class mentioned, respectively, while the existence of exosome-like vesicles released from the mitochondria has also been reported [13,14]. The contents of these EVs differ based on the cellular state [15], for example, apoptotic bodies are generated from the blebbing of the plasma membrane of cells undergoing apoptosis. Accordingly, microvesicles, also known as microparticles, represent a more heterogeneous subgroup of EVs and are produced from the plasma membranes of, for example, endothelial cells, red blood cells, and platelets. They also express TSG101 and retain markers of the cell of origin [16]. Exosomes, the main focus of this study, form from the inward budding of endosomes and are usually smaller than microvesicles, while both microvesicles and exosomes may be released by normal and tumour cells [13,17,18]. Consistently, exosomes may stably encapsulate various molecular cargo including DNA, mRNAs, miRNAs or proteins to target recipient cells and play essential roles in cell-to-cell communications [19,20]. These exosomes usually express markers including TSG101, heat shock protein 70 (HSP70), Alix 1, Tetraspanins (e.g., CD9, CD63, CD81, and CD82) and fibronectin, and akin to microvesicles will also contain molecular signatures of their cell of origin [21]. Accordingly, exosomes derived from NB tumours have been shown to contain a tumour-specific signature, are detectable in patient biofluids, and have a much higher DNA content than exosomes isolated from stromal cells [22]. This DNA can be greater than 10kb in length and cover the genomic DNA of all chromosomes representing the tumour genome [22], and may also contribute to disease progression, migration, metastasis and survival of cancer cells [15,23]. Hence, understanding the contributions of exosomes derived from NB or other cells of the tumour microenvironment (TME) to the biology of this disease would be of significant scientific interest. Figure 1A displays the general properties of exosomes derived from NB.

Taking a step forward from understanding the characteristics of exosomes, it stands to reason that their prognostic, diagnostic and therapeutic potential would be of importance to the scientific community. This is due to the relative convenience of extracting exosomes from peripheral blood to establish tumour genetic alterations rendering them a feasible molecular diagnostic tool for personalised medicine. In line with this objective, the application of whole exome sequencing of exosomal double-stranded DNA from NB patients for the detection of somatic mutations was explored and “exo-DNA” was shown to display tumour-specific genetic alterations including but not limited to *ALK*, *PHOX2B*, *TERT*, *PTPN11*, *SHANK2*, *KRAS*, and *FGFR1* [24] (Figure 1A). In addition, the authors also revealed that this DNA could be used to screen for acquired resistance to therapy including mutations in the RAS/MAPK pathways or alterations in *ALK* and *TP53* [24], highlighting the prognostic and diagnostic potential of this tool. Further to the diagnostic potential of NB-derived exosomes, their role in targeted therapy may also be significant. Therapeutic modalities in NB include resection that may be combined with chemotherapy and/or radiotherapy [25] and it is plausible that exosomes may impact both treatment response and potential relapse [26]. Given this background, the roles exosomes play in the biology, diagnosis, and treatment of NB have been addressed in this review (Figure 1B).

## 2. The Contribution of Exosomes to NB Biology and Management

### 2.1. Exosomes in NB Biology and Their Effect on the TME

#### 2.1.1. Exosomes in NB Biology

**Figure 2:**
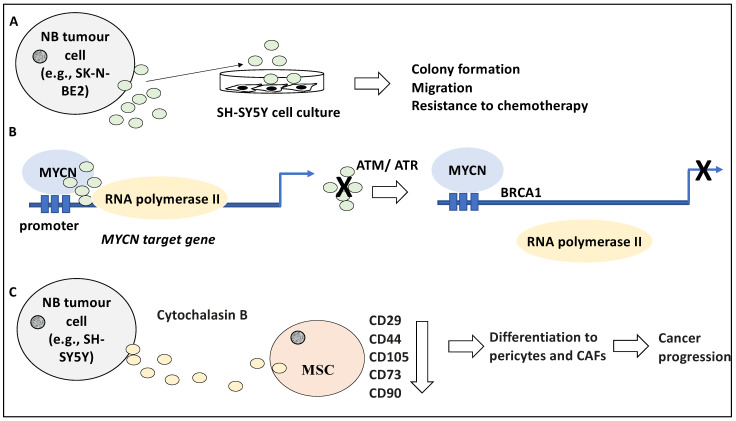
Exosomes affect NB biology and TME. (**A**) Exposing the SH-SY5Y cell line to exosomes derived from SK-N-BE2 led to elevated colony formation, migration, and resistance to chemotherapy-induced apoptosis. (**B**) MYCN recruited exoribonuclease-enclosed exosomes to the promoters of its targets involved in the cell cycle, thereby regulating the occupancy and elongation activity of RNA polymerase II. The depletion of these exosomes led to stalled replication forks and the activation of ATM and ATR, leading to the recruitment of BRCA1 and defective replication-transcription processes, respectively. (**C**) Cytochalasin B-induced membrane vesicles (CIMVs) released from SH-SY5Y cells treated with cytochalasin B were exposed to mesenchymal stem cells (MSCs) and this led to the reduced expression of MSC markers including CD29, CD44, CD105, CD73, and CD90. It is plausible that MSCs differentiated into pericytes and cancer-associated fibroblasts (CAFs) perhaps encouraging cancer progression.

The contribution of exosomes to the biology of NB may be understood better in light of the molecular content and secretion dynamics of these vesicles and how these may differ in NB tumours with aggressive phenotypes similar to that predicted for MYCN-amplified NB. For instance, Fonseka and colleagues have studied the proteomic profile of the proteins enclosed in exosomes derived from MYCN-amplified NB cells [27]. Using a nanoparticle tracking analysis (NTA) system, this study revealed that the isolated exosome from the NB cells was between 50–150 nm in diameter and that the MYCN-amplified NB cells generated much higher volumes of these exosomes compared to their non-MYCN amplified counterparts inclusive of SH-SY5Y [27]. Accordingly, 581 and 387 proteins were differentially present in exosomes derived from SK-N-BE2 and SH-SY5Y, respectively (a total of 968 proteins) [27]. For example, RAB proteins, involved in transport, were differentially present in exosomes isolated from these sources. Gene Ontology analysis revealed the enrichment of terms for transport, cell communication and signal transduction, as opposed to protein and nucleic acid metabolism and cell growth, for SK-N-BE2 and SH-SY5Y cells, respectively [27]. Interestingly, the treatment of SH-SY5Y cells with exosomes derived from SK-N-BE2 led to altered characteristics such as elevated colony formation potential, migration, and resistance to chemotherapy including Doxorubicin (Figure 2A). Inversely, the incubation of SH-SY5Y cells with exosomes derived from SK-N-BE2 with knocked-down MYCN reduced resistance to cell death by apoptosis [27]. Apart from revealing different molecular contents of MYCN-amplified and non-MYCN-amplified NB exosomes, this study suggested that MYCN-amplified NB-derived exosomes contain essential and transferable information that can instruct more aggressive behaviour in recipient non-MYCN-amplified NB cells. In our opinion, this is significant in outlining how cell-to-cell communications may alter cancer cell aggressiveness.

Given this interesting result, it was equally intriguing to understand if essential differences in the repertoire of exosomes also existed between primary and metastatic NB primary tissue. This question was answered by a study which profiled the proteomic signature of exosomes derived from primary NB and its bone marrow metastasis [28]. Accordingly, 15 and 6 proteins were found to be specific to primary and metastatic exosomes, respectively. From a functional viewpoint, the former group of proteins displayed roles in neuron development and extra-cellular matrix processes, compared to migration and mitochondrial activity for the latter group [28]. This study, therefore, revealed that the disease stage is reflected in exosome cargo, a significant finding for patient disease detection and progression screening. These two studies collectively conveyed the dynamic changes in the contents of these exosomes, which may reflect the relevant disease stage, and aggression, characteristics that seem, at least in part, transferable through these exosomes.

Stepping aside from the molecular contents of the exosomes and their implication in NB biology, exosomes may play an essential role in crucial cellular processes and functions including transcription factor occupancy and transcription elongation. For example, MYCN, a frequently amplified oncogene in NB, interacted with and recruited nuclear RNA exosomes (an exoribonuclease complex) to its target genes involved in cell-cycle processes such as S and G2 phase progression [29,30]. Consistently, MYCN, through these RNA exosomes, regulated the occupancy of RNA polymerase II by influencing “stalled RNA polymerase II at replication forks”, since the depletion of these exosomes led to both stalled forks and the slow RNA polymerase II elongation on cell cycle-associated genes, mainly MYCN targets [30]. Further, these exosomes prevented DNA damage namely double-strand breakage in MYCN-amplified NB cells. This was evident since the depletion of these exosomes led to the activation of ATM and BRCA1, dampening MYCN-related transcription through mRNA decapping mechanisms, and the activation of ATR leading to a defective replication-transcription process [30]. Hence, the RNA-enclosed exosomes were required for the MYCN-amplified NB cells, and NB cells depended on these exosomes to maintain elongation of transcription during the S and G2 phases and to avoid DNA damage, and defective transcription and replication processes [30] (Figure 2B). In conclusion, the exosomes recruited by MYCN served to advance the cell cycle-specific MYCN gene-regulatory programme; a significant finding concerning MYCN-amplified NB, a genetic alteration associated with poor patient outcomes [6,31,32]. In our opinion, perhaps the obvious step ahead may be formulating therapeutic strategies to specifically disrupt these exosomes and thereby break the link to MYCN-amplified NB progression.

#### 2.1.2. The Effect of Exosomes on the TME

Tumour-derived exosomes may influence and prime stromal and immune cells within the TME to further tumour growth and progression. For example, mesenchymal stem cells (MSCs) can migrate to the TME and it is thought that the differentiation of MSCs to pericytes and cancer-associated fibroblasts (CAFs), can form a tumour-supportive environment [33]. In line with this, the effect of the NB-derived EVs and their influence on the TME and stromal cells including MSCs was investigated in a study [33]. Initially, SH-SY5Y cells were exposed to cytochalasin B which disrupted the actin cytoskeleton and increased the generation and release of microvesicles from NB cells and were referred to as Cytochalasin B-induced membrane vesicles (CIMVs) [33]. These CIMVs were isolated using centrifugation and were analysed by microscopy and flow cytometry, revealing a size distribution of 100–1000 nm pertinent to both microvesicles and exosomes, and the expression of CD63, HSP70, and CD81 markers [33]. The effect of these CIMVs on MSCs was established by labelling and exposing these CIMVs to MSCs and it was revealed that after 24 h of co-culture, a small fraction of MSCs contained a large number of CIMVs while almost half contained smaller numbers of CIMVs suggesting a disparity in CIMV uptake by MSCs. The effect of the co-culture included a dose-dependent reduction in the expression of MSC markers such as CD29, CD44, CD105, CD73, and CD90, suggesting that the treatment may have altered the identity of the MSCs and perhaps directed their differentiation towards pericytes and CAFs encouraging cancer progression [33] (Figure 2C), although this aspect was not investigated. In agreement with the influence of NB tumour-derived exosomes on the TME, a study showed that bone marrow metastasis-derived NB cell lines secreted exosomes enclosing *miR-375*, which influenced osteogenic transcription factors [34]. This led to the expression of genes involved in bone differentiation, forming a tumour-supportive environment. Interestingly, higher levels of *miR-375* in patient circulating exosomes were linked to metastasis to the bone marrow and hence may be a useful biomarker for NB [34]. In our opinion, these were interesting findings since the exchange of vesicles between NB tumour cells and MSCs could have implications for tumour growth progression and the establishment of drug resistance, while it is equally plausible that vesicle-mediated cell-to-cell communications may be exploited for both diagnostic and therapeutic gain which will be addressed in the next sections.

### 2.2. Exosome-Enclosed Cargo Can Be Utilised as NB Biomarkers and Diagnostic Tools

**Figure 3:**
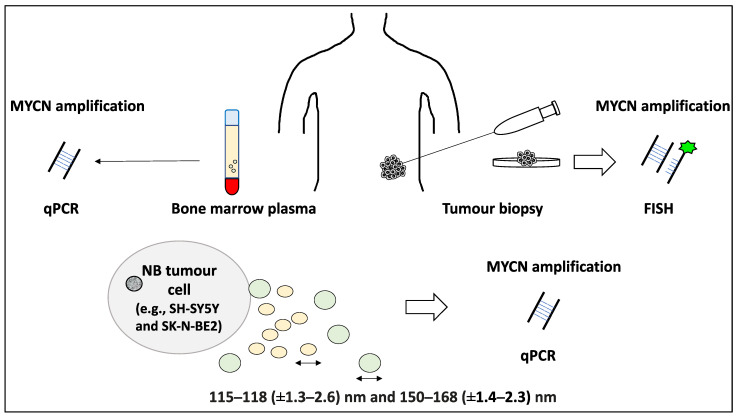
Diagnostic value of exosomes and microvesicles for NB patients for MYCN amplification status determination. EVs inclusive of exosomes and microvesicles (with diameters in the range of 115–168 and 150–168 nm, respectively) were isolated from SK-N-BE2 and SH-SY5Y cell culture supernatant and subjected to qPCR to detect MYCN amplification status, revealing that microvesicles yielded higher accuracy than exosomes presumably due to easier isolation and different biogenesis pathways. Concerning NB patient primary tissue samples, the standard diagnostic technique comprises tumour biopsy and detection of MYCN amplification by fluorescent in situ hybridisation (FISH), while it is possible to isolate microvesicles from the bone marrow plasma of patients to establish MYCN amplification status and this may yield largely accurate results.

The standard procedure for the detection of NB MYCN amplification is tumour biopsies from the primary site of presentation or metastatic sites, including the bone marrow and subsequent fluorescence in situ hybridisation (FISH) [18]. In many ways, the process of MYCN amplification status determination may delay stratification and the initiation of treatment; hence the identification of robust methods to reliably establish this essential diagnostic module is vital [35]. In a study aimed at establishing the diagnostic potential of exosomes and microvesicles for MYCN status establishment, these EVs were isolated from SK-N-BE2 and SH-SY5Y cell culture supernatants and subjected to ultracentrifugation, chromatography, NTA and Western blotting. For example, EV markers including HSP70, Alix 1 and TSG101 in addition to endosome markers including calnexin were utilised to confirm the presence of these EV particles in the lysates. The particles displayed a size distribution of 150–168 (±1.4–2.3) nm and 115–118 (±1.3–2.6) nm, which largely represent microvesicles and exosomes, respectively. Further, the total RNA of each EV class was subjected to qPCR and the MYCN status of the microvesicles rather than the exosomes were verifiable in multiple NB cell lines (i.e., positive and negative MYCN amplification status for SK-N-BE2 and SH-SY5Y, respectively) [18]. The authors contributed this difference in detection capacity to the biogenesis pathways and isolation methods for microvesicles and exosomes [18]. This study subsequently focused on comparing cytoplasmic MYCN RNA with that of their corresponding microvesicles and concluded that high levels of MYCN presence in the cytoplasm could get transferred to the microvesicles and consequently become more easily detectable [18]. Moreover, testing for MYCN amplification status in patient bone marrow plasma samples was also promising and 5/6 MYCN-amplified samples displayed MYCN+ microvesicles, while all 4 MYCN non-amplified samples displayed MYCN− microvesicle status, bearing a significant diagnostic potential for MYCN status establishment in these children [18,35] (Figure 3).

Noteworthy that the detection of tumour-derived components in addition to using tumour-derived exosome-based methods, can also be achieved through the detection of circulating tumour cells (CTCs) and of circulating-free DNA or cell-free DNA (cfDNA) [36]. With respect to the latter, a large body of studies has focused on using cfDNA retrieved from minimally invasive liquid biopsies from the patient circulatory system to detect genetic alterations and inform targeted treatment [37]. Concerning NB, the detection of specific genetic alterations including *MYCN* and *ALK* copy number status and clinically actionable *ALK* mutations including *ALK*-F1174L and *ALK*-R1275Q has been extensively reported [37,38,39,40,41,42]. For example, Lodrini and colleagues reported the application of droplet digital PCR (ddPCR) for the detection of *MYCN* and *ALK* copy number status in NB patient samples [39]. As a proof of principle experiment, the authors first established the *MYCN* and *ALK* gene copy number status in a panel of NB cell lines including IMR-32 and SH-SY5Y. Subsequently, cfDNA from the culture media of respective cell lines was also detected. As expected, the authors subsequently shifted to an animal model and detected cfDNA in the plasma of immunodeficient mice models xenografted with NB patient tumours [39]. Finally, the study reported the detection of *MYCN* and *ALK* copy number status in the plasma samples of 10 NB patients [39]. Interestingly, in a follow-up study, this group focused on detecting the mentioned genetic alterations in refractory and relapsed NB patient liquid biopsies as a longitudinal surveillance modality to detect resistant subclones and minimal residual disease. Accordingly, cfDNA from liquid biopsies and DNA from matched tumour samples of high-risk NB patients were collected and used to detect MYCN amplification and actionable *ALK* genetic alterations [42]. The results suggested superior early relapse detection capacity of liquid biopsies compared to other methods of somatic alteration detection in 2 patients. In addition, heterogeneity in relation to MYCN amplification and actionable *ALK* genetic alterations were detected in cfDNA samples for which such alterations were not detectable in their matched tumour tissue counterparts, suggesting the promising potential of liquid biopsies in capturing the heterogeneity of the tumour and relapsed samples [42]. In our opinion, these studies outline the significance of using exosomes and cfDNA in NB diagnosis of primary tumours, potential relapse, refraction and minimal residual disease that will have a bearing on both disease stratification and relevant treatment options. It would be interesting to conduct studies that may assess and compare the diagnostic accuracy and concordance between genetic alterations detectable using NB-tumour-derived exosomes and cfDNA and to discern whether these methods can be used in parallel and may indeed provide unique tumour-specific information.

### 2.3. Exosome-Enclosed Cargo Can Be Utilised for NB Treatment

#### 2.3.1. The Characteristics of Exosomes Used for NB Treatment

**Figure 4:**
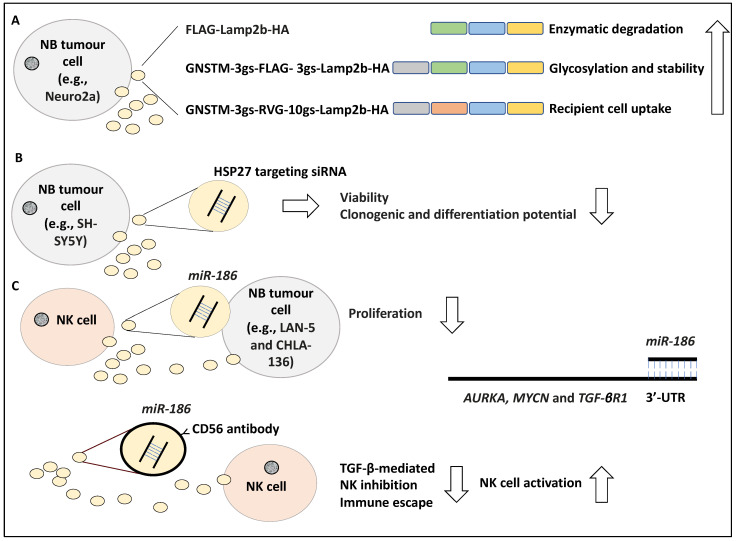
The stability of exosome peptides and exosome-based therapeutics. (**A**) Lamp2b, an exosome-associated protein fused to a FLAG tag (i.e., FLAG-Lamp2b-HA) was shown to be enzymatically cleaved by endosomal proteases; hence these peptides could not be detected in the recipient cells. The GNSTM tag was engineered to the N-terminus and displayed on exosomes which increased glycosylation, stabilising, and protecting the peptide from degradation. Further, the RVG tag in GNSTM-3gs-RVG-10gs-Lamp2b-HA construct displayed on exosomes was effectively received by recipient cells. (**B**) The siRNA-mediated knockdown of HSP27 in the SH-SY5Y cell line led to reduced viability, colony-forming, and differentiation potential. (**C**) Natural killer (NK) cells activated by IL-15 secreting exosomes containing *miR-186* were shown to induce cytotoxicity in MYCN-amplified NB cells, including LAN-5 and CHLA-136. *miR-186* directly targeted *AURKA*, *MYCN*, and *TGF-βR1* in NB cells, by binding to their 3′ untranslated region (3′-UTR). *miR-186* enclosed nanoparticles coated with CD56 antibody prevented TGF-β-triggered inhibition of NK cells, hence lowering immune escape and enhancing NK cell activation. The abbreviation “gs” stands for linkers.

It has been reported that exosomes can effectively target cells by displaying peptides on their surface, with the caveat that these peptides may be degraded. For example, in a study, the N-terminus FLAG peptide fused to an exosome-associated protein, Lamp2b (i.e., FLAG-Lamp2b-HA) was shown to be enzymatically cleaved by endosomal proteases, and hence this N terminus FLAG tag could not be detected in the harvested cells or exosomes, while the CD63 protein associated with exosomes was detected [43,44]. Huang and colleagues conducted this study in the Neuro2a NB cell line and to further address the stability of the N terminus FLAG-Lamp2b, they engineered a GNSTM amino acid sequence to the N terminus of the FLAG-Lamp2b-HA construct (e.g., GNSTM-3gs-FLAG-3gs-Lamp2b-HA in which “gs” stands for linkers) and displayed it on the exosomes. In evidence, the NST sequence attracted glycosylation while the flanking M and G amino acids further increased glycosylation occurrence, stabilising and protecting the peptide from cleavage and degradation [44,45]. Further, the fusion of a targeting peptide, RVG, fused to the N-terminus of Lamp2b, in addition to GNSTM, which was also displayed on exosomes allowed for effective uptake by recipient cells [46]. Effectively, the resulting GNSTM-3gs-RVG-10gs-Lamp2b-HA construct increased the levels of exosomal uptake compared to the negative controls including GNSTM-FLAG-Lamp2b-HA, assayed by PKH67 dye levels [44]. In conclusion, the authors showed that glycosylation of these peptides in addition to the inclusion of specific trafficking signal peptides prevented degradation, improved stability and targeted delivery of the exosomes [44] (Figure 4A). In our opinion, this study displayed how modifications to target peptides could effectively improve exosome-based therapies, a critical aspect to consider for drug delivery purposes [44].

#### 2.3.2. Viable Exosome-Based Treatment Options for NB

Differentiation induction is a viable treatment option for NB patients [47]. In a study, the role of HSP27 in regulating NB differentiation was investigated, whereby SH-SY5Y cell lines were exposed for 48 h to exosome-enclosed siRNAs targeting HSP27 and its effect on NB differentiation to neuron-like cells was established [48]. Accordingly, exosomes were characterised morphologically by electron microscopy and by the presence of CD63, while the uptake of the exosomes into the cells was monitored using immunofluorescence. Consistently, cell viability and clonogenic potential were assessed by MTT and colony-forming assays and NB differentiation was screened for using neural markers such as NeuN. Accordingly, viability, clonogenic, and differentiation potential were reduced due to the inhibition of HSP27 [48] (Figure 4B). This interesting study, in our opinion, revealed that exosome-based therapeutic molecule delivery is plausible and perhaps inducing HSP27 expression can encourage NB differentiation.

Immunotherapy has also been viewed as a viable treatment option for NB [49]. Several studies have introduced the interaction of NB with NK on the level of exosomes they exchange and how this may impact therapy. In a study, exosomes containing *miR-186* secreted by IL-15-activated natural killer (NK) cells were shown to induce cytotoxicity in MYCN-amplified NB cells, including LAN-5 and CHLA-136 [50]. In evidence, the low expression of *miR-186* in NB cells correlated with poor patient prognosis based on RNA-sequencing evidence retrieved from 498 primary patient tissues in which this miRNA was significantly downregulated [50]. In addition, the high expression of *miR-186* also correlated with the markers of activation of NK cells inclusive of NKG2D and DNAM-1, suggesting that perhaps higher expression of this miRNA may be linked to the presence of NK cells in the TME [50]. This group found that *miR-186* directly targeted *AURKA*, *MYCN* and *TGF-βR1* in NB cells, by binding to their 3′-UTR [50]. In addition, the ectopic expression of *miR-186* reduced the proliferation of LAN-5 and CHLA-136 cells. Consistently, the targeted delivery of *miR-186* reduced survival and migration in NB cells in mouse models. This was established using GD2/MYCN positive cell line CHLA-136 xenografted into the kidneys of the immunodeficient mouse model. Accordingly, *miR-186*-laden GD2 particles were successfully delivered to the tumour and this compound reduced tumour growth [50]. This group also tested the delivery of *miR-186* to NK cells and found that nanoparticles coated with CD56 antibody (targeting NK cells) containing *miR-186* prevented TGF-β-triggered inhibition of NK cells, hence *miR-186* reversed the TGF-β-based inactivation of NK cells and immune escape [50] (Figure 4C). In our opinion, the formulation of *miR-186* enclosed nanoparticles and exosomes could improve immunosurveillance of NB tumour cells and reduce tumour growth and proliferation [50].

**Figure 5:**
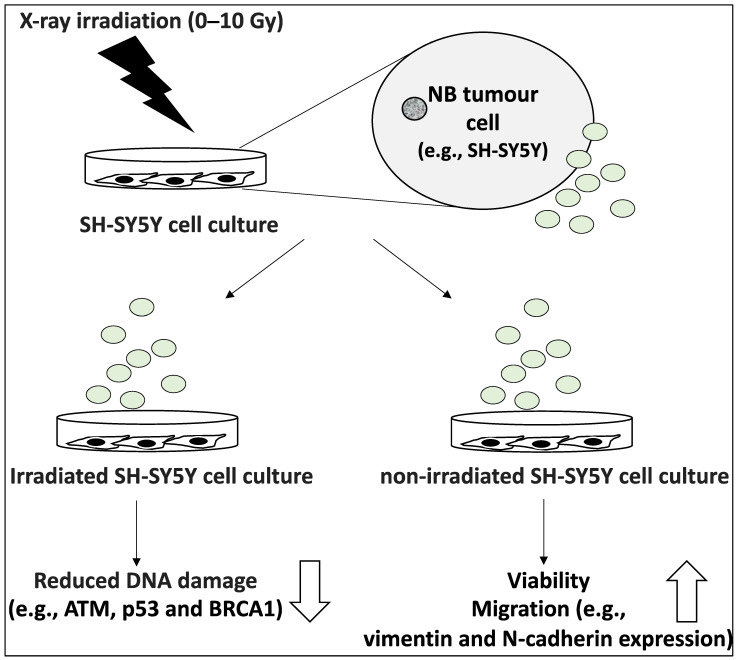
The characterisations of vesicles secreted by X-ray irradiated SH-SY5Y cells. SH-SY5Y cells were exposed to X-ray doses ranging from 0–10 Gy and secreted EVs were collected. These EVs were exposed to non-irradiated and irradiated SH-SY5Y cells. Non-irradiated SH-SY5Y cells exposed to these EVs displayed higher viability and increased motility evidenced by increased vimentin and N-cadherin levels. In irradiated cells treated with these EVs, the levels of DNA damage markers including ATM, p53 and BRCA1 were reduced and this reduction was inversely proportional to the irradiation dose given in the EV production experiment.

Radiotherapy represents one of the important treatment modalities of NB patient treatment options [25]. Standard doses include 21 Gy at 1.5 Gy per day and 21.6 Gy at 1.8 Gy per day but in some NB tumours, radiation therapy can lead to cellular stress setting in motion the release of an increased number of EVs with an altered cargo [51]. Radiotherapy can also lead to the rise of secondary tumours elsewhere in the body and this may be partially accounted for by the transfer of vesicles bearing vital pro-survival signals to non-irradiated cells [25,26]. In a study aiming to characterise the vesicles secreted by X-ray irradiated SH-SY5Y cells that serve to instruct non-irradiated NB cells, the EVs generated by the former were isolated and characterised [26]. Accordingly, the supernatant of NB cell lines exposed to X-ray doses ranging from 0–10 Gy was collected 3 h post-irradiation and was subsequently used to induce other SH-SY5Y cells in this study. The dose of X-ray did not alter the average size of the particles with a maximum size smaller than 160 nm in most cases; however, the number of these EVs increased with higher irradiation doses [26]. Further, the uptake of these EVs labelled with fluorescent dyes by recipient SH-SY5Y cells was monitored by imaging, and as expected, uptake was a function of incubation time [26]. Furthermore, the effects of these vesicles on viability, migration and DNA damage were established in non-irradiated and irradiated SH-SY5Y cells. For example, it was shown that exposure to EVs increased cell viability in non-irradiated SH-SY5Y cells by 20%, while these cells also showed reduced and increased p21 and pAKT protein levels compared to control cells, respectively, indicating cell cycle progression [26]. Further, cells treated with these EVs expressed vimentin and N-cadherin, suggesting a migratory phenotype [26]. Concerning the DNA damage, as expected, irradiation increased DNA damage levels established by DNA breaks and comet assays; however, pre-treating irradiated cells with these EVs (the vesicles secreted by X-ray irradiated SH-SY5Y) led to reduced DNA damage levels (including ATM, p53, and BRCA1), that was inversely proportional to the irradiation dose given to the donor cells. EV-induced repair may have occurred due to the accumulation of these cells in the G2/M stage to allow for essential repairs to take place [26] (Figure 5). In conclusion, these EVs induced proliferation, migration, and DNA repair in recipient NB cells. In our opinion, this study reiterated the impact of stress-induced exosome content and how this may ironically induce further disease progression and radioresistance.

## 3. Discussion

NB is the most prevalent extracranial childhood cancer and although rare may account for close to 15% of paediatric deaths due to cancer [5,6,52]. High-risk cases of this disease have a dismal 5-year survival rate of less than 50%. Multiple genetic alterations have been identified in NB and these may include MYCN amplification, which is the strongest predictor of poor outcomes in these children [5,6,52]. Given the complexity and potential aggression of this paediatric cancer, understanding various aspects of NB biology, diagnostic biomarkers and treatment options are of significant importance to the NB scientific community. One such promising line of investigation in recent years has been exosomes. Exosomes are shed by all cells including cancer cells and can be isolated from various sources such as patient biofluids [19,20]. These particles carry vital cargo and thereby transfer information from one cell to the next, hence may influence shaping the TME, in addition to tumour, immune and stromal cells behaviour that inadvertently will impact disease course, diagnosis and treatment [19,20]. Given this background, in this study, the role of exosomes in NB pathophysiology, their characteristics and how these properties may implicate diagnosis and treatment were catalogued and discussed.

In this study, using multiple examples, the characteristics of NB-derived exosomes and their contribution to the biology of NB within the TME were discussed [27,28,53]. For example, typically the combination of filtration and ultracentrifugation was used to isolate exosomes from NB cell lines including IMR-32, SH-SY5Y and HTLA-230. Subsequently, these exosomes would be subjected to light scattering analysis, NTA, and electron microscopy to determine the size distribution of these particles ranging from 68.05–83.66 nm [53], while the stability of these particles would be determined using various methods including Zeta-potential. Furthermore, the proteomic content of these exosomes could be established by 2D chromatography and mass spectrometry and typically included tetraspanins, HSPs, fibronectin, CD9, CD63 and CD81, CD133, CD147 and CD276, suggesting that these vesicles may have roles in cell proliferation, differentiation, defence and other biological processes [53]. In our opinion, it is imperative to correctly isolate and characterise exosome populations before conducting further investigative endeavours. Further to understanding the properties of NB cell-derived exosomes, the contribution of these exosomes to the biology of NB may be understood. Accordingly, in a study, the effect of exosomal *miR-17-5p* secreted from MYCN-amplified NB cells including SK-N-BE2 when co-cultured with non-MYCN NB cell lines such as SH-SY5Y was investigated [54]. *miR-17-5p* was directly linked to MYCN since MYCN directly bound the *miR-17-5p* promoter and its expression was 16-fold greater in SK-N-BE2 compared to SH-SY5Y cell line [55], while the co-culture of SK-N-BE2-derived exosomes with SH-SY5Y cells led to increase levels of *miR-17-5p* expression in SH-SY5Y cells [54]. The upregulation of *miR-17-5p* also increased the proliferative and migratory capacity of SH-SY5Y cells. The underlying mechanism was revealed as the activation of the PI3K/AKT pathway by *miR-17-5p* through PTEN inhibition, bringing into focus the significance of the intercellular communications that may indeed change the course of NB and how prognosis may be impacted through exosomes [54]. In our opinion, these studies have put into context that exosomes can alter the vital biology of the recipient NB cells within the TME, including proliferation, aggression, and disease progression.

Further to the impact of NB-derived exosomes on other NB cells, they can also impact other cells of the TME such as MSCs. We reviewed the CIMVs secreted from SH-SY5Y cells treated with Cytochalasin B led to the altered identity of MSCs to favour a tumour-friendly environment [33]. Interestingly, a similar premise applied to exosomes enclosing *miR-375* within the bone marrow NB metastasis, which changed the TME in a similar fashion [34], highlighting the impact of the exosomes derived from NB on manipulating the TME to more tumour-friendly environments.

Exosomes can also impact NB prognosis, diagnosis, and treatment. Concerning the prognostic value of exosomes, studies have revealed that it is possible to conduct genomic profiling of plasma-derived exosomal DNA using whole-exosome sequencing and microarray analysis of tumour tissue DNA, to predict progression from 4S to 4 stages in NB patient samples [56]. These data were sufficient to identify somatic genetic alterations accounting for NB tumour aggression including the loss of *KLRB1*, *MAPK3* and *FANCA* at the time of progression from 4S to 4, a determinantal switch in the patient’s prognosis [56]. From a diagnostic viewpoint, it has been shown that NB-derived exosomes expressed GD2 (ganglioside (disialoganglioside)) which is a significant biomarker of NB [53], suggesting the value of these particles to NB diagnosis. We also put into context the utility of tumour-derived exosomes and cfDNA for detecting crucial alterations to *ALK* and *MYCN* genes in NB patient primary and relapse samples [18,39,42], while comparative studies of the diagnostic accuracy of tumour-derived exosomes and cfDNA are still largely lacking.

Finally, we focused on the contribution of exosomes to NB treatment and their interaction with NK cells and the TME was reiterated [50]. Interestingly, exosomes derived from NK cells previously exposed to NB cells were shown to be greater in number than the exosomes produced by NK cells that were NB-naive [57]. Consistently, the former group of NK cells produced exosomes that displayed NK-specific receptors including NKG2D, CD56, and KIR2DL2 and were used to train naïve NK cells that then subsequently showed cytotoxicity towards NB cells assayed through both in vitro and in vivo systems [57]. This experiment has significant implications for priming NK cells and their exosomes to train naïve NK cells to enhance their anti-NB tumour reactivity and therefore can be exploited for tumour immunotherapy.

In conclusion, this study discussed the role of exosomes in contributing to NB biology, reshaping the TME, diagnosis and treatment that may indeed have a promising potential for the NB diagnostics and therapeutics field and ultimately improve the quality of life of NB sufferers.

## Abbreviations

ALT	Alternating length of telomeres
ATRX	Alpha Thalassemia mental Retardation-X linked
CAFs	Cancer-associated fibroblasts
CIMVs	Cytochalasin B-induced membrane vesicles
cfDNA	Circulating-free DNA or cell-free DNA
ddPCR	Droplet digital PCR
EVs	Extracellular vesicles
FISH	fluorescent in situ hybridisation
GD2	Ganglioside (disialoganglioside)
HSP70	Heat shock protein 70
IDRF	Image-defined risk factors
INSS	International neuroblastoma staging system
INRG	International neuroblastoma risk group
INRGSS	Neuroblastoma risk group staging system
MSCs	Mesenchymal stem cells
NB	Neuroblastoma
NTA	Nanoparticle tracking analysis
TERT	Telomere reverse transcriptase
TME	Tumour microenvironment
TMM	Telomere maintenance mechanisms
3′-UTR	3′-untranslated region
